# Individualizing Follow-Up Strategies in High-Grade Soft Tissue Sarcoma with Flexible Parametric Competing Risk Regression Models

**DOI:** 10.3390/cancers12010047

**Published:** 2019-12-21

**Authors:** Maria Anna Smolle, Michiel van de Sande, Dario Callegaro, Jay Wunder, Andrew Hayes, Lukas Leitner, Marko Bergovec, Per-Ulf Tunn, Veroniek van Praag, Marta Fiocco, Joannis Panotopoulos, Madeleine Willegger, Reinhard Windhager, Sander P. D. Dijkstra, Winan J. van Houdt, Jakob M. Riedl, Michael Stotz, Armin Gerger, Martin Pichler, Herbert Stöger, Bernadette Liegl-Atzwanger, Josef Smolle, Dimosthenis Andreou, Andreas Leithner, Alessandro Gronchi, Rick L. Haas, Joanna Szkandera

**Affiliations:** 1 Department of Orthopaedics and Trauma, Medical University of Graz, 8036 Graz, Austria; 2 Department of Orthopaedic Surgery, Leiden University Medical Centre, 2333 ZA Leiden, The Netherlands; 3 Department of Surgery, Fondazione IRCCS Istituto Nazionale dei Tumori, 20133 Milan, Italy; 4 University Musculoskeletal Oncology Unit, Mount Sinai Hospital, University of Toronto, Toronto, ON M5G IX5, Canada; 5 Department of Surgery, Royal Marsden Hospital NHS Foundation Trust, London SW3 6JJ, UK; 6 Sarcoma Centre, HELIOS-Klinikum Berlin-Buch, 13125 Berlin, Germany; 7 Institute of Mathematics, Leiden University Medical Centre, 2333 ZA Leiden, The Netherlands; 8 Medical Statistics, Department of Biomedical Data Science, Leiden University Medical Centre, 2333 ZA Leiden, The Netherlands; 9 Princess Máxima Center for Pediatric Oncology, Trial and Data Center, 3584 CT Utrecht, The Netherlands; 10 Department of Orthopaedics and Traumatology, Medical University of Vienna, 1090 Vienna, Austria; 11 Department of Surgery, The Netherlands Cancer Institute, 1066 CX Amsterdam, The Netherlands; 12 Division of Clinical Oncology, Department of Medicine, Medical University of Graz, 8036 Graz, Austria; 13 Institute of Pathology, Medical University of Graz, 8010 Graz, Austria; 14 Institute for Medical Informatics, Statistics and Documentation, Medical University of Graz, 8036 Graz, Austria; 15 Sarcoma Centre, HELIOS Klinikum Bad Saarow, 15526 Bad Saarow, Germany; 16 Department of Radiotherapy, Leiden University Medical Centre, 2333 ZA Leiden, The Netherlands; 17 Department of Radiotherapy, The Netherlands Cancer Institute Amsterdam, 1066 CX Amsterdam, The Netherlands

**Keywords:** soft tissue sarcoma, follow-up, flexible parametric competing risk regression model, local recurrence, distant metastasis

## Abstract

Currently, patients with extremity soft tissue sarcoma (eSTS) who have undergone curative resection are followed up by a heuristic approach, not covering individual patient risks. The aim of this study was to develop two flexible parametric competing risk regression models (FPCRRMs) for local recurrence (LR) and distant metastasis (DM), aiming at providing guidance on how to individually follow-up patients. Three thousand sixteen patients (1931 test, 1085 validation cohort) with high-grade eSTS were included in this retrospective, multicenter study. Histology (9 categories), grading (time-varying covariate), gender, age, tumor size, margins, (neo)adjuvant radiotherapy (RTX), and neoadjuvant chemotherapy (CTX) were used in the FPCRRMs and performance tested with Harrell-C-index. Median follow-up was 50 months (interquartile range: 23.3–95 months). Two hundred forty-two (12.5%) and 603 (31.2%) of test cohort patients developed LR and DM. Factors significantly associated with LR were gender, size, histology, neo- and adjuvant RTX, and margins. Parameters associated with DM were margins, grading, gender, size, histology, and neoadjuvant RTX. C-statistics was computed for internal (C-index for LR: 0.705, for DM: 0.723) and external cohort (C-index for LR: 0.683, for DM: 0.772). Depending on clinical, pathological, and patient-related parameters, LR- and DM-risks vary. With the present model, implemented in the updated Personalised Sarcoma Care (PERSARC)-app, more individualized prediction of LR/DM-risks is made possible.

## 1. Introduction

Patients with high-grade extremity soft tissue sarcoma (eSTS) are at risk of developing local recurrences (LR) and even more so of developing distant metastases (DM) after having undergone surgical resection of the primary tumor [1,2,3,4,5]. These rates differ substantially per size, grade, and subtype [6]. Close follow-up regimens are currently used in order to detect LR and DM at stages where they are still potentially treatable by re-resection or metastasectomy, respectively [7]. There is no clear consensus when, by what means, and how often to perform follow-up in eSTS patients, with many centers and guidelines having introduced a heuristic approach: for the first 3 years after surgery, patients would be checked three or four times a year, then biannually for the following two years and annually thereafter [8,9].

Imposing all eSTS patients on these strict follow-up regimens has raised public, scientific, and health economic concerns over the last years. Numerous factors interact with the risk of developing LR or DM, such as histological STS-subtypes, surgical margins, tumor size, grade, administration of neo(adjuvant) radiotherapy (RTX) or chemotherapy (CTX), and patient-derived factors [1,2,3,4,10,11,12]. Consequently, the current approach of “one-size-fits-all” may not account for the unequal risk of recurrence in the heterogeneous eSTS population, involving an excessive number of surveillance imaging, possibly leading to unnecessary delivery of imaging-induced radiation exposure, and the inherent burden for radiology departments, as well as inappropriately refraining from it, a high number of outpatient visits and financial costs and emotional stress for each individual patient [13]. However, an evaluation of prognostic factors for LR and DM taking into consideration the time-varying rate for the occurrence of events in a multicenter cohort, including important patient-(i.e., age, gender), tumor-(e.g., size, grade, histological subtype), and treatment-related features (e.g., margins, (neo)adjuvant CTX/RTX), is currently missing. 

Therefore, the aim of the present study was to estimate and validate two models predicting risks of LR and DM over the first 5 years of follow-up by applying flexible parametric competing risk regression modeling in a large, multicenter cohort of patients with primary localized high-grade eSTS. The results have been implemented into the Personalised Sarcoma Care (PERSARC)-app [14] for Individualized Sarcoma Care and follow-up. 

## 2. Results

Patients had undergone surgery with curative intent between January 1994 and October 2014 for the test cohort and between January 2000 and December 2013 for the validation cohort, respectively. There was a slight male predominance (*n* = 1038; 53.8%) and the median patient age was 59 years (interquartile range (IQR): 44.7–70 years). With 55.8%, 17.9%, and 13.9%, most tumors in the test cohort were located in the thigh (*n* = 1078), upper arm (*n* = 346), and lower leg (*n* = 268), while the lower arm (*n* = 142), the foot or toes (*n* = 65), and the hand or fingers (*n* = 32) were affected in 7.3%, 3.4%, and 1.7%, respectively. Further clinicopathological features for both the test and validation cohort are listed in Table 1.

Five- and 10-year overall survival (OS) was 73.6% (95%CI: 71.3–75.7) and 62.7% (95%CI: 59.8–65.5) in the test cohort. In the validation cohort, 5- and 10-year OS were 64.9% (95%CI: 61.8–67.8) and 52.9% (95%CI: 48.9–56.8), respectively. Gender, tumor size, histological subtype (except for angiosarcoma/vascular sarcoma (*p* = 0.127) and dedifferentiated/pleomorphic liposarcoma (*p* = 0.254), margins, neoadjuvant and adjuvant RTX, as well as adjuvant CTX (all *p* < 0.05) had a significant influence on risk of LR in the stepwise backward selection of the Fine and Gray model. Grading as a time-dependent effect was kept in the model (*p* = 0.108), while age (*p* = 0.082) and neoadjuvant CTX (*p* = 0.214) were excluded. Consequently, gender, grading, tumor size, neoadjuvant and adjuvant RTX, histological subtype, and adjuvant CTX were included in the flexible parametric competing risk regression model (Table 2).

**Figure 1:**
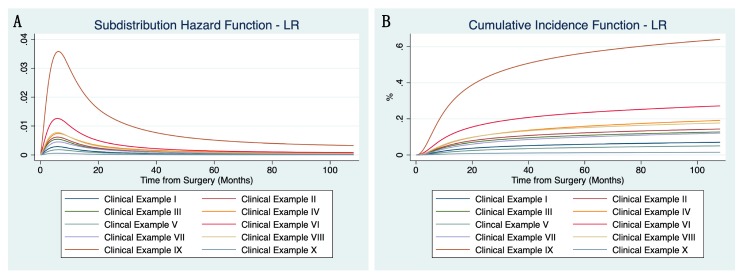
Subdistribution hazard function (**A**) and cumulative incidence function (**B**) of the flexible parametric competing risk regression model for local recurrence using ten clinical examples (constellation of parameters shown in Tables S1 and S2).

The subdistribution hazard and cumulative incidence functions for LR using ten clinical examples are shown in Figure 1A,B (definition of these examples found in Table S1, together with estimated conditional risks of LR). As an example, a male patient with a G2 myxofibrosarcoma sized 10 cm, with contaminated resection margins (R1/2), no neoadjuvant or adjuvant RTX, and no adjuvant CTX, has a significantly increased risk of developing LR, especially within the first 15 months of follow-up (=clinical example IX). On the other hand, a male patient with a 6 cm large, G3 synovial sarcoma, resected with clear margins (R0), without adjuvant CTX or (neo-)adjuvant RTX, has a moderate LR risk during the first 15 months, and an estimated low risk thereafter (=clinical example VIII).

In the stepwise backward selection of the Fine and Gray model for distant metastasis (DM) histological subtype (except for myxofibrosarcoma (*p* = 0.641), angiosarcoma/vascular sarcoma (*p* = 0.067) and dedifferentiated/pleomorphic liposarcoma (*p* = 0.592), grading, tumor size, gender, margins, and neoadjuvant RTX (all *p* < 0.05) were significantly associated with development of metastases. Age (*p* = 0.852), adjuvant RTX (*p* = 0.116), neoadjuvant CTX (*p* = 0.095), and adjuvant CTX (*p* = 0.536) were excluded via stepwise backward selection. Thus, histological subtype, grading, tumor size, margins, gender, and neoadjuvant RTX were included in the flexible parametric competing risk regression model (Table 3). 

**Figure 2:**
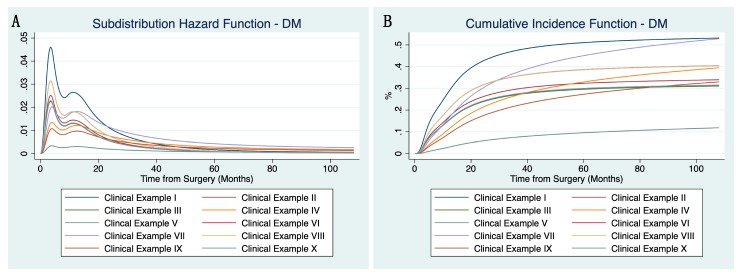
Subdistribution hazard function (**A**) and cumulative incidence function (**B**) of the flexible parametric competing risk regression model for distant metastasis using the same ten clinical examples as in Figure 1A,B (constellation of parameters shown in Tables S1 and S2).

In Figure 2A,B, subdistribution hazard and cumulative incidence functions for DM (using the same ten clinical examples as in Figure 1A,B) are shown. Once again referring to clinical examples IX (male, myxofibrosarcoma, G2, 10 cm, R1/2-margins, no neoadjuvant RTX) and VIII (male, synovial sarcoma, G3, 6cm, R0-margins, no neoadjuvant RTX), risk of DM is lower in clinical example IX in comparison to clinical example VI, while LR-risks are just the opposite, highlighting the importance of an individualized follow-up strategy.

The conditional risks of these ten clinical examples changing over time estimated based on the models presented above are provided in Table S1 for LR and Table S2 for DM. Conditional risks for all possible combinations of prognostic factors may be estimated and have been implemented in the updated version of the PERSARC app. 

**Figure 3:**
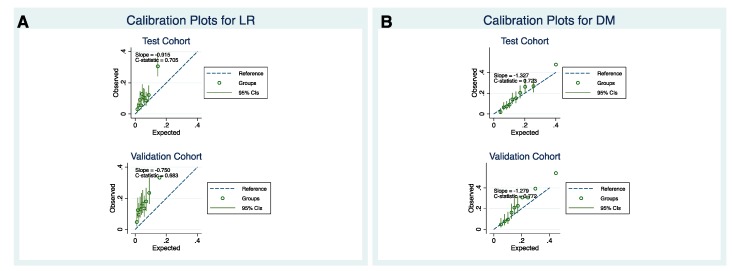
Calibration plots for the flexible parametric competing risk regression model regarding local recurrence (**A**) and distant metastasis (**B**) for the test (top) and validation cohort (bottom).

The Harrell C index for LR was equal to 0.705 and 0.683 for the internal and external cohort, respectively. For DM, Harrell C statistics was equal to 0.723 for the internal cohort and 0.772 for the external cohort. Calibration plots for LR (Figure 3A) using test and validation cohort showed that the LR model tended to underestimate the actual patient risk, especially in the validation cohort. On the other hand, calibration plots for DM with test and validation cohort (Figure 3B) showed very good model calibration.

## 3. Discussion

In the present retrospective multicenter cohort study, flexible parametric competing risk regression modeling was applied in order to estimate individual three-to-six-month risks for local recurrence and distant metastasis during the first 5 years of follow-up in patients undergoing curative resection for high-grade extremity soft tissue sarcoma. It offers an evidence-based opportunity to individually schedule follow-up visits instead of adhering to calendar-based guidelines [8,9]. The number of radiological investigations for assessing disease status, especially after R0 resections and taking histological subtype into account, could be significantly restricted, reducing patient- and healthcare burden. The advantage of using flexible parametric competing risk regression models to estimate LR- and DM-risks in eSTS-patients is based on the fact that these rates strongly vary upon time (i.e., they do not constantly increase or decrease). To overcome this issue, flexible parametric competing risk regression models represent the baseline distribution function as a restricted cubic spline function of log time instead of a linear function of log time [15]. Moreover, it allows smooth estimation of both the cause-specific hazard rates and cumulative incidence functions. Both models performed well at internal and external calibration, with c-indexes comparable to previously published studies [14,16].

One of the limitations of the present study is its retrospective design, resulting in possible selection biases regarding diagnosis, treatment, and follow-up of patients included, due to slightly differing policies at the respective centers. By incorporating these factors in the statistical models, we aimed at reducing this bias. Moreover, during the study period, several histological STS-subtypes were reclassified (i.e., malignant fibrous histiocytoma to undifferentiated pleomorphic sarcoma). At some, but not all, participating centers, all histological diagnoses had been reevaluated by pathologists and, if applicable, reclassified according to the current classification systems. In order to limit the impact of this limitation, we only included patients treated in tertiary reference sarcoma centers with experienced and dedicated sarcoma pathologists.

Another limitation of the present study is that the models were developed based on patient cohorts from experienced, tertiary tumor centers. This implies that generalizability of the predicted risks to patients not treated at such centers has to be questioned. Moreover, considering that we did only include patients with high-grade (G2/3), primary eSTS who had undergone surgery with curative intent, the risks estimated are not applicable to patients with low-grade disease or metastases at initial presentation. Furthermore, estimated risks of the current models should be applied with caution after patients have already developed an event (i.e., LR or DM) during follow-up. Due to the retrospective design of the study, not all variables could be ascertained in every patient, thus potentially reducing the statistical power. However, it can be assumed that in this large patient collective, missing data may have little to no bias to the conclusions made, wherefore cases with missing information on clinical and/or pathological variables were not excluded [17].

As outlined in the introduction, current follow-up strategies follow a heuristic approach, with 3- to 4-months intervals for the first three years, followed by biannual check-ups until the end of the 5th year and annual appointments thereafter [8,9].

In clinical practice, it is not only of interest to estimate a patient’s cumulative risk after a specific period of time but also to know about the conditional risks from one follow-up appointment to the next, in case no event had occurred. We addressed this question by calculating conditional risks for LR and DM depending on different, clinically relevant, examples. Notably, the present model allows risk prediction at any constellation of variables, which are at the moment included in the updated PERSARC app. This app allows the patient’s individualized risk of LR and DM to be estimated by entering relevant prognostic parameters, such as histological subtype, tumor size, and resection margins. With the estimated event-risk in time, physicians and patients may decide together when the next follow-up examination should be scheduled. In light of the heterogeneity of eSTS with part significantly differing outcomes, estimated event risks would facilitate planning of an individualized follow-up protocol for each patient.

Arbitrary thresholds of 4% for LR and 2% for DM were chosen in the present study to be of clinical “relevance”, considering that LR is usually detected during clinical examination or even noticed by patients themselves, while DM (most commonly to the lungs) require visualization by chest x-ray or thoraci computed tomography (CT) scan [18,19]. However, thresholds should be changed on patients’ preference and clinical significance.

Previously published studies have well-investigated risks of LR, DM, and overall survival (OS) in large, retrospective cohorts of patients with eSTS [14,16,20,21]. The nomogram for OS by Kattan et al. [21] in 2002 and the two more recent nomograms for DM and OS by Callegaro et al. [16] published in 2016 added significant value to predict individual patient risks. Both studies used Cox-regression models as the basis for their nomograms. In comparison to Cox-regression models, flexible parametric competing risk regression models have a major advantage; while the Cox-regression models only estimate the relative effects (i.e., hazard rates), flexible parametric competing risk regression models estimate the baseline hazard using restricted cubic splines [22]. The cumulative incidence functions of LR and DM predicted from flexible parametric competing risk regression models demonstrate the clear variance in event rates. By applying a flexible parametric competing risk regression model, we aimed at incorporating non-constant hazards, time-varying covariates, and death as the competing event in order to obtain a robust, comprehensible, and accurate prediction of individual patient risks. Moreover, with the clinical examples provided, the risk peaks during the first year of follow-up is clearly visible. Although appointments may be safely skipped in some patients due to very low risks of LR and/or DM, others would benefit from closer follow-up intervals.

Potentially due to the application of the present statistical model, interesting results emerged: Female gender was independently associated with a significantly lower risk of LR and DM. An association between gender and overall survival (OS) has been observed by Maretty-Nielsen et al. and Wu et al. [2,23]. However, an association between gender and LR-free as well as DM-free survival has not been described thus far [24]. Moreover, tumor grading, which is a well-known prognostic factor of LR, was not significantly associated with an altered risk in the current flexible parametric competing risk regression model. This may be explained by the fact that patients with usually fast-growing, highly-aggressive G3 tumors will present with LR at early time points, while in those with relatively slower-growing G2 tumors, LR is most probably detected at a later date. This hypothesis is corroborated by the fact that grading did not meet the proportional hazards assumption, wherefore it was treated as a time-varying covariate. On the other hand, another recently published multicenter study for grade III eSTS did not incorporate grade II in the multivariate model for OS [14]. The current model has broader applicability as it also incorporates patients with grade II eSTS. Additionally, margins as classified in the current study only divide “clear” from “contaminated” margins, not taking into consideration that histological subtypes with infiltrative growth pattern as undifferentiated pleiomorphic sarcoma (UPS) and myxofibrosarcoma potentially require broader margins to markedly reduce LR-risk [25]. Thus, unsurprisingly, also in the current flexible parametric competing risk regression model for LR, these histological subtypes showed significantly higher LR-risks in comparison to other histologies.

## 4. Materials and Methods

In this retrospective multicenter study, 1931 consecutive patients with primary nonmetastatic high-grade (G2/3) eSTS managed with surgery at a curative intent were included in the test cohort, with patient information deriving from prospectively maintained STS databases at 5 participating tertiary sarcoma referral centers. Patients with missing information on oncological follow-up (i.e., development of LR/DM) had to be excluded (*n* = 42). Extremity STS were defined as tumors from the shoulder to the fingers (=upper limb) and from the pelvic girdle, excluding intrapelvic STS, to the foot (=lower limb). The validation cohort consisted of 1085 patients with identical inclusion criteria as for the test cohort from two independent tertiary sarcoma referral centers. As described above, patient monitoring after surgery usually followed a standardized approach with clinical examination, radiography using chest X-ray (CXR) or chest CT-scan (chest-CT) for control of DM and sonography or magnetic resonance imaging (MRI) for control of LR.

Demographic variables (patient age at diagnosis, gender), tumor-related parameters (tumor size, depth, location, grading, histological subtype), treatment (histological margins, (neo)adjuvant CTX/RTX), and outcome variables (date of LR or DM, date of death/last follow-up) were reported. Histological resection margins were divided into “clear” margins (=R0) and “contaminated” margins (=R1/2), as classification and definition of margin status have changed over time [26,27,28]. Histological subtypes were classified into 9 categories, with myxoid liposarcoma as the reference, compliant with previous studies and the current World Health Organisation (WHO) classification (Table 1) [6,16]. The FNCLCC grading system (Fédération Française des Centres de Lutte Contre le Cancer/French Federation of Centres for the Fight against Cancer) was used to categorize tumors into either intermediate (=G2) or high-grade (G3) [29]. (Neo-)adjuvant RTX and CTX had been administered in case a high risk of LR or DM had been anticipated by the multidisciplinary tumor board, according to locally preferred guidelines, LR was defined as a radiologically and/or histologically confirmed tumor recurrence. DM must have been confirmed by radiography (sonography, MRI, CXR, chest-CT) and/or histologically. In the case of pulmonary nodules without subsequent surgical exploration, an increase in size of the suspected metastasis must have been present. Patient, tumor, and treatment-related factors were ascertained using medical records, histological reports, and prospectively maintained databases at the respective centers.

Ethics approval was obtained in each participating center. The study was performed according to the Declaration of Helsinki and approved by the Ethics Committee of the Medical University of Graz, Austria (IRB-approval-number: 31-046 ex 18/19; date of approval: 24 May 2019).

### Statistical Analysis

We focused on the first five years of follow-up to predict the conditional risk at the usually scheduled follow-up times (every 3 months from 1st to 3rd year; every 6 months in 4th and 5th year), i.e., the risk of experiencing an event at X + Y, given that the patient has not developed an event before X months. The variables age and tumor size were centered at their mean value in order to allow prediction at the average in case variables were not specified upon calculation. We used the Royston and Parmar approach to fit a flexible parametric competing risk regression model in order to estimate the risk of LR and DM, with death as the competing event [30]. In this model, the baseline distribution is modeled as a restricted cubic spline function of log time [15,22]. Splines constitute flexible mathematical functions defined by piecewise polynomials together with distinct constraints, ensuring that the overall curve is smooth [22]. A feature of restricted cubic splines as used in the present model is that the fitted function is forced to be linear before the first and after the last knot [31]. As automatic stepwise backward selection of variables is currently not available for the flexible parametric competing risk regression model, variable selection for the LR and DM models was based on a stepwise backward procedure using a multivariable Fine and Gray model [32]. Variables with a *p*-value < 0.05 were excluded from the model, except for histology, where all subtypes were kept in the analyses. The LR and DM models were fit on the log cumulative subdistribution hazard scale, directly modeling the cause-specific cumulative incidence function. Grading was incorporated in the model as a time-dependent effect for LR and DM, as it did not meet the proportional hazards assumption. The number of knots of the flexible parametric competing risk regression model for LR and DM was chosen based on the lowest AIC (=Akaike Information Criterion) after fitting several models with knots from 0 to 5. For the flexible parametric competing risk regression model estimating the risk of LR, two knots turned out as most accurate, while for the model predicting the risk of DM, four knots were used (with no internal knots for grading as a time-dependent covariate). Cumulative incidence functions were estimated based on the defined models. Conditional risks at the 3–6-months intervals were calculated based on the cumulative incidence functions of the flexible parametric competing risk regression model. Threshold was set to 4% for LR, considering that they are often palpable and diagnosed during the clinical examination or by patients themselves [18]. On the other hand, a 2% threshold for DM was chosen, as DM (and most commonly lung metastases) can only safely be diagnosed by chest X-ray or CT-scans of the thorax [19]. Model discrimination was tested using the Harrell C index, estimating the probability of concordance between observed and predicted outcomes. A value of 0.5 indicates no predictive discrimination, while a value of 1.0 indicates a perfect separation of patients with different outcomes [33]. Furthermore, calibration plots were compiled to assess model calibration in the test and validation cohort.

## 5. Conclusions

In conclusion, the present study provides a model to individually predict patient’s LR and DM risks during follow-up, applying a flexible parametric competing risk regression approach. These models are at the moment being included in the updated version of the PERSARC app for Individualized Sarcoma Care and follow-up. Although a risk-threshold of 4% for LR and 2% for DM was chosen in the present study, the “optimal” threshold upon which an individual patient should undergo imaging with MRI, chest-CT, or CXR, is still subjected to experts’ opinion and should be further discussed with patients concerned.

## Figures and Tables

**Table 1:** Patient characteristics and clinical, pathological, and treatment-related parameters.

Variables	Test Cohort (*n* = 1931)			Validation Cohort (*n* = 1085)		
		N (%)	Missing (%)	N (%)	Missing (%)	*p*-Value *
**Age** (continuous; years; median + IQR)		59 (44.7–70)	45 (2.3)	61 (47–74)	0 (0.0)	**<0.0001**
**Gender**	Male	1038 (53.8)	0 (0.0)	615 (56.7)	0 (0.0)	0.121
	Female	893 (46.2)		470 (43.3)		
**Tumor Location**	Upper Extremity	520 (26.9)	1 (0.05)	312 (28.8)	0 (0.0)	0.285
	Lower Extremity	1410 (73.1)		773 (71.2)		
**Depth**	Epifascial	518 (26.9)	2 (0.1)	291 (26.8)	0 (0.0)	0.984
	Subfascial	1411 (73.1)		794 (73.2)		
**Tumor Size** (continuous; cm; median + IQR)		7 (4–11)	30 (1.6)	7.5 (5–12)	5 (0.5)	**0.026**
**Histology**	Myxoid liposarcoma	222 (11.6)	16 (0.8)	111 (10.2)	0 (0.0)	**<0.0001**
	MPNST	83 (4.3)		43 (4.0)		
	Myxofibrosarcoma	451(23.6)		104 (9.6)		
	Synovial Sarcoma	174 (9.1)		79 (7.3)		
	UPS	325 (17.0)		375 (34.6)		
	Angiosarcoma/Vascular Sarcoma	22 (1.1)		20 (1.8)		
	Dedifferentiated/Pleomorphic Liposarcoma	141 (7.4)		85 (7.8)		
	Leiomyosarcoma	221 (11.5)		118 (10.9)		
	Others	276 (14.4)		150 (13.8)		
**Grading**	G2	719 (37.8)	30 (1.6)	382 (35.8)	19 (1.8)	0.282
	G3	1182 (62.2)		684 (64.2)		
**Margins**	R0	1494 (77.4)	0 (0.0)	768 (70.8)	0 (0.0)	**<0.0001**
	R1/2	437 (22.6)		317 (29.2)		
**CTX**	No	1408 (73.0)	1 (0. 05)	1039 (95.8)	0 (0.0)	**<0.0001**
	Neoadjuvant	262 (13.6)		40 (3.7)		
	Adjuvant	209 (10.8)		6 (0.6)		
	Neoadjuvant + Adjuvant	51 (2.6)		0 (0.0)		
**RTX**	No	619 (32.9)	50 (2.6)	335 (30.9)	0 (0.0)	**<0.0001**
	Neoadjuvant	303 (16.1)		460 (42.4)		
	Adjuvant	956 (50.8)		275 (25.4)		
	Neoadjuvant + Adjuvant	3 (0.2)		15 (1.4)		
**Follow-up** (continuous; months; median + IQR)		50 (23.3–95)	11 (0.6)	56 (21–91)	0 (0.0)	0.254

* *p*-values calculated with Wilcoxon rank sum test for continuous variables, with chi2-test for binary and categorical variables. *p*-values in bold are considered statistically significant. Abbreviations: CTX = chemotherapy. IQR = interquartile range. MPNST = Malignant Peripheral Nerve Sheat Tumor. RTX = radiotherapy. UPS = Undifferentiated pleiomorphic sarcoma.

**Table 2:** Estimated coefficients along with their 95% confidence intervals for local recurrence.

Variables		Coefficient	95%-CI		*p*-value
			*Lower*	*Upper*	
**Local Recurrence**					
Gender	Male	1			**0.011**
	Female	0.698	0.529	0.921	
Grading	G2	1			0.199
	G3	0.816	0.598	1.113	
Tumor size		1.026	1.004	1.049	**0.019**
Margins	R0	1			**<0.001**
	R1/R2	2.761	2.021	3.774	
Histology	Myxoid Liposarcoma	1			
	MPNST	4.227	1.837	9.729	**0.001**
	Myxofibrosarcoma	4.156	2.056	8.400	**<0.001**
	Synovial Sarcoma	3.116	1.429	7.014	**0.005**
	UPS	3.373	1.620	7.025	**0.001**
	Angiosarcoma/Vascular Sarcoma	3.316	0.981	12.341	0.074
	Dedifferentiated/Pleomorphic Liposarcoma	1.727	0.719	4.143	0.221
	Leiomyosarcoma	2.779	1.294	5.966	**0.009**
	Others	2.385	1.123	5.065	**0.024**
Neoadjuvant RTX	No	1			**<0.001**
	Yes	0.298	0.178	0.494	
Adjuvant RTX	No	1			**0.001**
	Yes	0.603	0.447	0.814	
Adjuvant CTX	No	1			**0.008**
	*Yes*	*1.711*	*1.154*	*2.538*	
Restricted cubic spline 1		2.104	1.851	2.392	**<0.001**
Restricted cubic spline 2		1.332	1.230	1.442	**<0.001**
Restricted cubic spline 3		0.980	0.937	1.026	0.391
Restricted cubic spline for time-dependent effect of grading		0.944	0.813	1.096	0.449
Constant		0.048	0.024	0.097	**<0.001**
Death					
Gender	Male	1			**0.005**
	Female	0.736	0.595	0.910	
Grading	G2	1			**<0.001**
	G3	2.215	1.655	2.964	
Tumor size		1.065	1.048	1.081	**<0.001**
Margins	R0	1			0.296
	R1/R2	1.153	0.883	1.057	
Histology	Myxoid Liposarcoma	1			
	MPNST	1.205	0.664	2.187	0.540
	Myxofibrosarcoma	1.208	0.795	1.836	0.375
	Synovial Sarcoma	1.461	0.888	2.404	0.136
	UPS	1.150	0.753	1.758	0.517
	Angiosarcoma/Vascular Sarcoma	4.729	2.335	9.580	**<0.001**
	Dedifferentiated/Pleomorphic Liposarcoma	1.420	0.863	2.338	0.167
	Leiomyosarcoma	2.154	1.402	3.309	**<0.001**
	Others	1.516	0.975	2.356	0.065
Neoadjuvant RTX	No	1			**0.007**
	Yes	1.543	1.127	2.111	
Adjuvant RTX	No	1			0.296
	Yes	1.145	0.888	1.476	
Adjuvant CTX	No	1			**0.022**
	Yes	0.679	0.488	0.946	
Restricted cubic spline 1		4.220	3.393	5.250	**<0.001**
Restricted cubic spline 2		1.487	1.329	1.663	**<0.001**
Restricted cubic spline 3		0.965	0.921	1.011	0.139
Restricted cubic spline for time-dependent effect of grading		0.714	0.580	0.889	**0.002**
Constant		0.050	0.032	0.078	**<0.001**

*p*-values in bold are considered statistically significant. CI = Confidence interval. RTX = radiotherapy. CTX = chemotherapy. UPS = undifferentiated pleomorphic sarcoma. MPNST = Malignant Peripheral Nerve Sheat Tumour.

**Table 3:** Estimated coefficients along with their 95% confidence intervals for distant metastasis.

		Coefficient	95%-CI		*p*-Value
			Lower	Upper	
**Distant Metastasis**					
**Gender**	**Male**	1			**<0.001**
	Female	0.720	0.605	0.857	
**Grading**	G2	1			**<0.001**
	G3	1.737	1.412	2.136	
**Tumor size**		1.069	1.056	1.083	**<0.001**
**Margins**	R0	1			**0.006**
	R1/R2	1.347	1.087	1.669	
**Histology**	Myxoid Liposarcoma	1			
	MPNST	1.825	1.158	2.875	**0.009**
	Myxofibrosarcoma	1.064	0.750	1.508	0.729
	Synovial Sarcoma	1.986	1.343	3.976	**0.001**
	UPS	1.445	1.033	2.022	**0.032**
	Angiosarcoma/Vascular Sarcoma	2.016	1.022	3.797	**0.043**
	Dedifferentiated/Pleomorphic Liposarcoma	1.209	0.786	1.861	0.387
	Leiomyosarcoma	2.689	1.900	3.797	**<0.001**
	Other	1.835	1.293	2.604	**0.001**
**Neoadjuvant RTX**	No	1			**0.005**
	Yes	1.351	1.097	1.663	
**Restricted cubic spline 1**		2.928	2.591	3.308	**<0.001**
**Restricted cubic spline 2**		1.458	1.374	1.547	**<0.001**
**Restricted cubic spline 3**		0.965	0.926	1.006	0.096
**Restricted cubic spline 4**		1.040	1.020	1.062	**<0.001**
**Restricted cubic spline 5**		0.995	0.982	1.008	**0.427**
**Restricted cubic spline for time-dependent effect of grading**		0.723	0.640	0.817	**<0.001**
**Constant**		0.108	0.078	0.149	**<0.001**
**Death**					
**Gender**	Male	1			0.864
	Female	0.968	0.666	1.407	
**Grading**	G2	1			**0.018**
	G3	1.873	1.116	3.145	
**Tumor size**		1.023	0.997	1.050	0.087
**Margins**	R0	1			0.198
	R1/R2	1.378	0.846	2.244	
**Histology**	Myxoid Liposarcoma	1			
	MPNST	2.506	0.844	7.442	0.098
	Myxofibrosarcoma	3.136	1.325	7.418	**0.009**
	Synovial Sarcoma	0.600	0.150	2.416	0.472
	UPS	1.781	0.714	4.443	0.216
	Angiosarcoma/Vascular Sarcoma	11.165 *	3.507 *	35.542 *	<0.001 *
	Dedifferentiated/Pleomorphic Liposarcoma	3.331	1.259	8.812	**0.015**
	Leiomyosarcoma	1.798	0.675	4.782	0.241
	Other	2.408	0.963	4.782	0.060
**Neoadjuvant RTX**	No	1			**0.048**
	Yes	0.541	0.295	0.993	
**Restricted cubic spline 1**		3.604	2.494	5.211	**<0.001**
**Restricted cubic spline 2**		1.270	1.060	1.523	**0.010**
**Restricted cubic spline 3**		0.952	0.863	1.049	0.316
**Restricted cubic spline 4**		0.953	0.908	1.001	0.057
**Restricted cubic spline 5**		0.974	0.569	1.199	0.097
**Restricted cubic spline for time-dependent effect of grading**		0.826	0.569	1.199	0.314
**Constant**		0.010	0.004	0.025	**<0.001**

*p*-values in bold are considered statistically significant; CI = Confidence interval. RTX = radiotherapy. CTX = chemotherapy. UPS = undifferentiated pleomorphic sarcoma. * too few events.

## Supplementary Materials

The following are available online at https://www.mdpi.com/2072-6694/12/1/47/s1, Table S1: Conditional Risks for Local Recurrence–Threshold 4%; Table S2: Conditional Risks for Distant Metastasis–Threshold 2%.
