# Supplementary material for: Individualizing Follow-Up Strategies in High-Grade Soft Tissue Sarcoma with Flexible Parametric Competing Risk Regression Models
Source: Cancers (Basel). 2019 Dec 21;12(1):47. doi: 10.3390/cancers12010047 (PMC7017264; doi:10.3390/cancers12010047)
Supplement: Supplementary file 1 [file cancers-12-00047-s001.pdf]

cancers

MDPI

# Supplementary Material

*Article*

# Individualising follow-up strategies in high-grade soft tissue sarcoma with flexible parametric competing risk regression models.

**Table S1.** Conditional Risks for Local Recurrence – Threshold 4%.

| Interval (months) | 0-3 | 3-6 | 6-9 | 9-12 | 12-15 | 15-18 | 18-21 | 21-24 | 24-27 | 27-30 | 30-33 | 33-36 | 36-42 | 42-48 | 48-54 | 54-60 |
|---|---|---|---|---|---|---|---|---|---|---|---|---|---|---|---|---|
| **Clinical Example I** | 0.3 | 0.8 | 0.8 | 0.7 | 0.5 | 0.3 | 0.3 | 0.3 | 0.3 | 0.2 | 0.2 | 0.2 | 0.3 | 0.3 | 0.2 | 0.2 |
| UPS, G3, 12cm, male, R0, neoadj. RTX, no adj. RTX, no adj. CTX | 1.1 | | 1.5 | | 0.9 | | 0.6 | | 0.5 | | 0.4 | | 0.6 | | 0.4 | |
| **Clinical Example II** | 0.7 | 1.7 | 1.7 | 1.4 | 1.1 | 0.9 | 0.7 | 0.6 | 0.5 | 0.5 | 0.4 | 0.8 | 0.7 | 0.5 | 0.5 | 0.4 |
| Myxofibrosarcoma, G3, 8cm, female, R1/2, neoadj. RTX, no adj. RTX, no adj. CTX | 2.4 | | 3.1 | | 2.0 | | 1.4 | | 1.0 | | 1.0 | | 1.2 | | 1.0 | |
| **Clinical Example III** | 0.7 | 1.5 | 1.5 | 1.2 | 1.0 | 0.8 | 0.7 | 1.2 | 0.5 | 0.4 | 0.4 | 0.3 | 0.6 | 0.5 | 0.5 | 0.4 |
| Synovial sarcoma, G3, 6cm, female, R0, no neoadj. RTX, no adj. RTX, no adj. CTX | 2.2 | | 2.8 | | 1.8 | | 1.0 | | 0.9 | | 0.7 | | 1.1 | | 0.8 | |
| **Clinical Example IV** | 0.1 | 2.0 | 2.1 | 1.8 | 1.5 | 1.2 | 1.0 | 0.9 | 0.8 | 0.7 | 0.6 | 0.6 | 1.0 | 0.8 | 0.7 | 0.7 |
| Leiomyosarcoma, G2, 4cm, male, R0, no neoadj. RTX, no adj. RTX, no adj. CTX | 2.8 | | 3.9 | | 2.6 | | 1.9 | | 1.4 | | 1.2 | | 1.8 | | 1.4 | |
| **Clinical Example V** | 0.2 | 0.5 | 0.5 | 0.4 | 0.4 | 0.3 | 0.2 | 0.2 | 0.2 | 0.2 | 0.2 | 0.1 | 0.2 | 0.2 | 0.2 | 0.2 |
| | 0.7 | | 1.0 | | 0.7 | | 0.5 | | 0.4 | | 0.3 | | 0.4 | | 0.3 | |

| | | | | | | | | | | | | | | | | |
|---|---|---|---|---|---|---|---|---|---|---|---|---|---|---|---|---|
| Myxoid liposarcoma, G2, 3cm, female, R0, no neoadj. RTX, no adj. RTX, no adj. CTX | | | | | | | | | | | | | | | | |
| **Clinical Example VI** | 1.5 | 3.5 | 3.5 | 2.9 | 2.3 | 1.8 | 1.5 | 1.3 | 1.1 | 1.0 | 0.9 | 0.8 | 1.3 | 1.1 | 1.0 | 0.9 |
| Dediff. Liposarcoma, G3, 9cm, male, R1/2, no neoadj. RTX, no adj. RTX, no adj. CTX | 4.9 | | 6.3 | | 4.1 | | 2.8 | | 2.1 | | 1.6 | | 2.5 | | 1.8 | |
| **Clinical Example VII** | 0.5 | 1.2 | 1.3 | 1.1 | 0.9 | 0.7 | 0.6 | 0.5 | 0.5 | 0.4 | 0.4 | 0.3 | 0.6 | 0.5 | 0.4 | 0.4 |
| Leiomyosarcoma, G2, 6cm, female, R1/2, neoadj. RTX, no adj. RTX, no adj. CTX | 1.7 | | 2.4 | | 1.6 | | 1.1 | | 0.9 | | 0.7 | | 1.1 | | 0.9 | |
| **Clinical Example VIII** | 0.9 | 2.1 | 2.2 | 1.8 | 1.4 | 1.1 | 0.9 | 0.8 | 0.7 | 0.6 | 0.5 | 0.5 | 0.8 | 0.7 | 0.6 | 0.5 |
| Synovial sarcoma, G3, 6cm, male, R0, no neoadj. RTX, no adj. RTX, no adj. CTX | 3.1 | | 3.9 | | 2.5 | | 1.7 | | 1.3 | | 1.0 | | 1.5 | | 1.1 | |
| **Clinical Example IX** | 4.0 | 9.4 | 9.9 | 8.4 | 6.8 | 5.7 | 4.8 | 4.1 | 3.6 | 3.3 | 2.9 | 2.7 | 4.6 | 4.0 | 3.5 | 3.2 |
| Myxofibrosarcoma, G2, 10cm, male, R1/2, no neoadj. RTX, no adj. RTX, no adj. CTX | 12.9 | | 17.4 | | 12.1 | | 8.7 | | 6.8 | | 5.5 | | 8.4 | | 6.6 | |
| **Clinical Example X** | 0.1 | 0.1 | 0.2 | 0.1 | 0.1 | 0.1 | 0.1 | 0.1 | 0.1 | 0.04 | 0.04 | 0.04 | 0.1 | 0.1 | 0.04 | 0.04 |
| Myxoid liposarcoma, G3, 12cm, female, R0, neoadjuvant RTX, no adj. RTX, no adj. CTX | 0.2 | | 0.3 | | 0.2 | | 0.1 | | 0.1 | | 0.1 | | 0.1 | | 0.1 | |

***Legend***: *Conditional risks for local recurrence according to the currently applied follow-up program for ten different clinical examples using the test cohort. Shaded areas highlight follow-up intervals exceeding the threshold of 4% for local recurrence. The first row for each example depicts conditional risks at every 3 months, whilst in the second row, the predicted conditional risks in 6-month-intervals are shown.*

**Table S2.** Conditional Risks for Distant Metastasis – Threshold 2%.

| Interval (months) | 0-3 | 3-6 | 6-9 | 9-12 | 12-15 | 15-18 | 18-21 | 21-24 | 24-27 | 27-30 | 30-33 | 33-36 | 36-42 | 42-48 | 48-54 | 54-60 |
|---|---|---|---|---|---|---|---|---|---|---|---|---|---|---|---|---|
| **Clinical Example I** | 5.8 | 11.2 | 7.4 | 7.5 | 7.2 | 5.9 | 4.6 | 3.6 | 2.9 | 2.4 | 2.0 | 1.7 | 2.6 | 1.9 | 1.5 | 1.1 |
| UPS, G3, 12cm, male, R0, neoadj. RTX | 17.8 | | 14.3 | | 12.7 | | 7.8 | | 5.2 | | 3.6 | | 4.5 | | 2.6 | |
| **Clinical Example II** | 3.2 | 6.3 | 4.1 | 4.3 | 4.0 | 3.3 | 2.5 | 2.0 | 1.6 | 1.3 | 1.1 | 1.9 | 1.4 | 1.0 | 0.8 | 0.6 |
| Myxofibrosarcoma, G3, 8cm, female, R1/2, neoadj. RTX | 9.3 | | 8.1 | | 7.1 | | 4.5 | | 2.9 | | 2.0 | | 2.5 | | 1.4 | |
| **Clinical Example III** | 2.9 | 5.7 | 3.7 | 3.7 | 3.6 | 2.9 | 2.3 | 1.8 | 1.4 | 1.2 | 1.0 | 0.8 | 1.3 | 0.9 | 0.7 | 0.6 |
| Synovial sarcoma, G3, 6cm, female, R0, no neoadj. RTX | 8.4 | | 7.3 | | 6.4 | | 4.0 | | 2.6 | | 1.8 | | 2.2 | | 1.3 | |
| **Clinical Example IV** | 1.4 | 3.7 | 3.1 | 3.4 | 3.5 | 3.2 | 2.7 | 2.4 | 2.1 | 1.9 | 1.7 | 1.5 | 2.7 | 2.3 | 2.0 | 1.8 |
| Leiomyosarcoma, G2, 4cm, male, R0, no neoadj. RTX | 5.1 | | 6.4 | | 6.6 | | 5.0 | | 3.9 | | 3.2 | | 4.9 | | 3.8 | |
| **Clinical Example V** | 0.4 | 0.9 | 0.8 | 0.9 | 0.9 | 0.8 | 0.7 | 0.6 | 0.5 | 0.5 | 0.4 | 0.4 | 0.7 | 0.6 | 0.5 | 0.5 |
| Myxoid liposarcoma, G2, 3cm, female, R0, no neoadj. RTX | 1.3 | | 1.6 | | 1.7 | | 1.3 | | 1.0 | | 0.8 | | 1.3 | | 1.0 | |
| **Clinical Example VI** | 4.0 | 7.8 | 5.1 | 5.2 | 5.0 | 4.1 | 3.1 | 2.5 | 2.0 | 1.6 | 1.4 | 2.5 | 1.8 | 1.3 | 1.0 | 0.8 |
| Dediff. Liposarcoma, G3, 9cm, male, R1/2, no neoadj. RTX | 11.5 | | 10.0 | | 8.8 | | 5.5 | | 3.6 | | 2.9 | | 3.1 | | 1.6 | |
| **Clinical Example VII** | 2.1 | 5.5 | 4.6 | 5.1 | 5.2 | 4.7 | 4.1 | 3.5 | 3.1 | 2.8 | 2.5 | 2.3 | 4.0 | 3.4 | 3.0 | 2.7 |
| Leiomyosarcoma, G2, 6cm, female, R1/2, neoadj. RTX | 7.5 | | 9.4 | | 9.7 | | 7.5 | | 5.8 | | 4.8 | | 7.3 | | 5.6 | |
| **Clinical Example VIII** | 4.0 | 7.8 | 5.1 | 5.2 | 5.0 | 4.1 | 3.1 | 2.5 | 2.0 | 1.6 | 1.4 | 1.1 | 1.8 | 1.3 | 1.0 | 0.7 |
| Synovial sarcoma, G3, 6cm, male, R0, no neoadj. RTX | 11.5 | | 9.8 | | 8.8 | | 5.5 | | 3.6 | | 2.5 | | 3.1 | | 1.8 | |
| **Clinical Example IX** | 1.1 | 3.0 | 2.5 | 2.7 | 2.8 | 2.5 | 2.2 | 1.9 | 1.7 | 1.5 | 1.4 | 1.2 | 2.1 | 1.8 | 1.6 | 1.4 |
| Myxofibrosarcoma, G2, 10cm, male, R1/2, no neoadj. RTX | 4.1 | | 5.1 | | 5.3 | | 4.0 | | 3.2 | | 2.6 | | 3.9 | | 3.0 | |

| | | | | | | | | | | | | | | | | |
|---|---|---|---|---|---|---|---|---|---|---|---|---|---|---|---|---|
| **Clinical Example X** | 3.0 | 5.8 | 3.7 | 3.8 | 3.7 | 3.0 | 2.3 | 1.8 | 1.5 | 1.2 | 1.0 | 0.8 | 1.3 | 1.0 | 0.7 | 0.6 |
| Myxoid liposarcoma, G3, 12cm, female, R0, neoadjuvant RTX | 8.5 | | 7.4 | | 6.5 | | 4.1 | | 2.6 | | 1.8 | | 2.3 | | 1.3 | |

***Legend***: *Conditional risks for distant metastasis according to the currently applied follow-up program for ten different clinical examples using the test cohort. Shaded areas highlight follow-up intervals exceeding the threshold of 2% for distant metastasis. The first row for each example depicts conditional risks at every 3 months, whilst in the second row, the predicted conditional risks in 6-month-intervals are shown.*
